# Advances in electrical impedance tomography-based brain imaging

**DOI:** 10.1186/s40779-022-00370-7

**Published:** 2022-02-28

**Authors:** Xi-Yang Ke, Wei Hou, Qi Huang, Xue Hou, Xue-Ying Bao, Wei-Xuan Kong, Cheng-Xiang Li, Yu-Qi Qiu, Si-Yi Hu, Li-Hua Dong

**Affiliations:** 1 Department of Radiation Oncology and Therapy, The First Hospital of Jilin University, 130021 Changchun, China; 2 Jilin Provincial Key Laboratory of Radiation Oncology and Therapy, The First Hospital of Jilin University, Changchun, 130021 China; 3 NHC Key Laboratory of Radiobiology, School of Public Health, Jilin University, Changchun, 130021 China; 4 CAS Key Laboratory of Bio-Medical Diagnostics, Suzhou Institute of Biomedical Engineering and Technology, Chinese Academy of Sciences, Suzhou, 215163 Jiangsu China

**Keywords:** Electrical impedance tomography (EIT), Brain diseases, Tissue impedance, Microelectrode array

## Abstract

Novel advances in the field of brain imaging have enabled the unprecedented clinical application of various imaging modalities to facilitate disease diagnosis and treatment. Electrical impedance tomography (EIT) is a functional imaging technique that measures the transfer impedances between electrodes on the body surface to estimate the spatial distribution of electrical properties of tissues. EIT offers many advantages over other neuroimaging technologies, which has led to its potential clinical use. This qualitative review provides an overview of the basic principles, algorithms, and system composition of EIT. Recent advances in the field of EIT are discussed in the context of epilepsy, stroke, brain injuries and edema, and other brain diseases. Further, we summarize factors limiting the development of brain EIT and highlight prospects for the field. In epilepsy imaging, there have been advances in EIT imaging depth, from cortical to subcortical regions. In stroke research, a bedside EIT stroke monitoring system has been developed for clinical practice, and data support the role of EIT in multi-modal imaging for diagnosing stroke. Additionally, EIT has been applied to monitor the changes in brain water content associated with cerebral edema, enabling the early identification of brain edema and the evaluation of mannitol dehydration. However, anatomically realistic geometry, inhomogeneity, cranium completeness, anisotropy and skull type, etc., must be considered to improve the accuracy of EIT modeling. Thus, the further establishment of EIT as a mature and routine diagnostic technique will necessitate the accumulation of more supporting evidence.

## Background

Electrical impedance tomography (EIT) is a non-invasive and non-radiation technique that allows imaging of the electrical impedance variations inside a volume of interest. EIT is realized by the mathematical reconstruction of electrical impedance data collected from an external electrodes array [1]. EIT is not only an alternative to traditional imaging modalities, but can also address some limitations of other imaging modalities (Table 1). For example, functional connectivity photoacoustic tomography, a functional imaging technique based on the acoustic detection of optical absorption from the optical excitation of tissue chromophores [2], enables noninvasive imaging of resting-state functional connectivity in the mouse brain with a large field of view and high spatial resolution [3], but has lower resolution and penetration depth than EIT [4]. High-resolution ultrasound provides a good axial resolution of up to 400 μm, but has low soft-tissue contrast [5]. Computer tomography (CT) and magnetic resonance imaging (MRI) ideally provide high-resolution body maps of solid tumors, and positron emission tomography (PET) imaging of cancer depends on metabolic activity rather than size [6]. However, CT and PET require the use of ionizing radiation with potential carcinogenic effects, which renders these methods less suitable for screening asymptomatic individuals. Furthermore, MRI acquisition takes a long time and requires a high degree of patient cooperation. In addition, these imaging devices are very expensive, and the portability of hardware is limited. In contrast, EIT extracts the electrical impedance characteristics closely related to tissue function, and can detect prospective changes that occur before organizational structural changes. EIT offers several advantages, including low cost, real-time imaging, operational simplicity, and minimal side effects [7, 8]. Therefore, the use of EIT as an auxiliary functional imaging modality for bedside monitoring is growing, with good application prospects.

**Table 1:** Comparison of characteristics of brain imaging technologies

Technique	Mechanism of operation	Cost	Wearable	Operability	Side effects	Precision
EIT	Electrical impedance in tissue	Low	Yes	Maneuverable	No	Low
fcPAT	Optical excitation and acoustic detection	Low	Yes	Maneuverable	No	High
EEG	Electrical activity of brain cells	Low	Yes	Maneuverable	No	Low
X-ray	Low energy X-rays	Moderate	No	Professional	Radiation	Moderate
CT	X-rays, computer processing and conversion	High	No	Professional	Radiation	High
MRI	Magnetic field and pulsating radio waves	High	No	Professional	Time-consuming	High
PET	Gamma rays emitted by tracer substance	High	No	Professional	Radiation	Moderate

*EIT* electrical impedance tomography, *fcPAT* functional connectivity photoacoustic tomography, *EEG* electroencephalography, *CT* computed tomography, *MRI* magnetic resonance imaging, *PET* positron emission tomography

EIT originated from archaeological geophysics research more than a century ago and has been used medically for approximately 40 years [9]. After decades of research and innovation, the clinical utility of EIT for monitoring lung function and breast cancer has been demonstrated [10, 11], and research of its potential use in detecting epileptic seizure onset zones [12], stroke [13], and in the evaluation dehydration treatment for cerebral edema [14] is in progress. This review briefly introduces the history, basic principles, algorithms, and system composition of EIT. Further, current and past research on the use of EIT in epilepsy, stroke, brain injury, and brain edema are reviewed, and representative studies on this technique are summarized. Finally, the limitations of EIT are outlined, and the prospects of applying magnetic resonance EIT (MREIT) and microelectrode array EIT for the diagnosis and treatment of brain diseases are discussed.

## Basic principles

The basic principle of EIT involves injecting a weak excitation current into a specific body region and imaging changes in the distribution of electrical parameters in the region of interest by measuring voltage signals on the boundary electrode positioned on the body surface [8]. Different body tissues possess different resistivity, which may also be altered in diseased states. As such, physiological and pathological changes in internal impedance can be captured with EIT imaging [15]. Single-source and single-frequency excitation is the most common data acquisition method in EIT because of its wide application and easy operation [16].

In the adjacent drive, current running through neighboring electrodes is injected and voltage is measured at the remaining electrodes [17]. An opposing pair of electrodes (such as 1–9) in the electrode current incentive method is known as the opposite or polar method drive [17], in this method, the current is injected through a pair of opposite electrodes and voltage differences are measured on the remaining electrodes, relative to the voltage reference electrode, which corresponds to that adjacent to the current-injecting electrode. This process is repeated until the current has been injected between all pairs of electrodes. Different current drive excitation methods produce distinct internal electric field distributions, which affect the signal detection ability and image reconstruction quality [18].

Static reconstruction reconstructs the absolute distribution of electrical impedance. In contrast, dynamic reconstruction involves the measurement of two frames of data at different times, and a subsequent reconstruction of the difference in electrical impedance between these timepoints. Due to the effects of model errors and the amount of calculation, the application of static reconstruction in clinical practice has been challenging. However, model errors in dynamic reconstruction are effectively eliminated by the calculation of differences, which minimizes the influence of model errors and system accuracy requirements. Notably, the volume of calculations is small, which makes this approach easy to implement. Accordingly, dynamic reconstruction has obvious advantages in the field of medical bedside monitoring. Electrical impedance imaging technology has been employed in the rapid detection and monitoring of breast cancer, acute respiratory distress syndrome, stroke, and other diseases using dynamic reconstruction methods [19–21]. Other EIT technologies, such as multi-frequency EIT [22], Lorentz force EIT [23], magnetic induction EIT [24], rotating EIT [25], etc., are emerging according to different application scenarios and requirements.

## EIT algorithms

Electrical impedance imaging employs different modes of current excitation [2, 26–28]. The mode of current excitation from two adjacent electrodes is called adjacent excitation, and that from two relative electrodes (such as 1–9 electrodes) is called opposite excitation. Although different modes of current excitation generate different electric field distributions inside, under the condition of low frequency and weak current, EIT can meet the conditions of a quasi-stable field regardless of the excitation mode.

In isotropic media, the EIT field equation can be described using the Laplace equation:

$$\nabla \cdot [\gamma (x,y)\nabla \Phi (x,y)] = 0$$

where $\nabla$ is the vector differential operator, $\Phi (x,y)$ is the potential distribution in the field, and $\gamma (x,y)$ is the impedance distribution in the field, which consists of two parts:

$$\gamma (x,y) = \sigma (x,y) + j\omega \varepsilon (x,y)$$

where $\sigma (x,y)$ is the pure conductivity; $\varepsilon (x,y)$ is the dielectric constant; and *ω* is the angular frequency of the electric field. In electrical impedance imaging measurement, the excitation source frequency generally ranges 10–100 kHz. Under this frequency range, the influence of the dielectric constant can be ignored, and the following equation can be obtained:

$$\nabla \cdot \sigma \nabla \Phi = 0$$

The corresponding boundary conditions include an imposed boundary condition and a natural boundary condition, they are as follows:

Impose boundary condition:

$$\Phi = \overline{\Phi }$$

Natural boundary conditions:

$$\sigma \frac{\partial \Phi }{{\partial n}} = \overline{\varphi }$$

where $\overline{\Phi }$ and $\overline{\varphi }$ correspond to the voltage and current density of the boundary region, respectively. When the internal conductivity distribution and boundary conditions (such as excitation current) are known, the spatial potential distribution can be calculated employing the finite element method and other methods [29].

According to the Geselowitz sensitivity relationship [30], the region is discretized, and a uniform electrical impedance distribution is set in each small unit. The forward problem of dynamic electrical impedance imaging under current excitation can be described as follows:

$$v = S\rho$$

where $v$ is the measured voltage change vector,$\rho$ is the discrete electrical impedance change vector, and *S* is the sensitivity coefficient matrix. The above equation indicates that if the change in electrical impedance is known, the change in boundary measurement voltage can be calculated to complete the calculation of the EIT forward problem [31].

Image reconstruction is a process of reconstructing the internal resistivity distribution from the boundary voltage signal. If the boundary measurement voltage change is known, the internal electrical impedance change can be obtained by inverting the forward problem:

$$\rho = S^{ - 1} v$$

where $S^{ - 1}$ is the inverse of ${\varvec{S}}$. In most cases, the measured values can only be obtained by measuring at several independent points in the continuous domain; however, theoretically, there are infinitely many model parameters in the domain. Therefore, the inverse problem is poorly posed. To overcome this problem, the inverse problem must be solved by regularization; the regularization term is used to improve the ill-posed nature of the problem, such as with the damped least-squares reconstruction method:

$$\overset{\lower0.5em\hbox{$\smash{\scriptscriptstyle\frown}$}}{\rho } = (S^{T} S + \lambda R)^{ - 1} S^{T} v$$

in which the $R$ matrix is the regularized matrix and $\lambda$ is the regularized parameter.

Given different application requirements, such as imaging speed, boundary retention, uniformity, resolution, etc., scholars have made full use of various sources of prior information, and have proposed many different algorithms, such as L1, L2, and Lp regularization [32–37]. Borsic et al. [33] presented the use of primal–dual interior-point methods (PDIPMs) for efficiently using total variation (TV) as a regularization function in EIT; through numerical experiments and the reconstruction of medical data, they showed that TV regularization algorithms produced sharper images than those with quadratic regularization algorithms, and that these algorithms can be successfully applied to real data from medical experiments. Jin et al. [34] developed a sparse reconstruction algorithm, motivated by a Tikhonov function that incorporated a sparsity-promoting L1-penalty term; the results indicated that the proposed technique could yield quantitatively acceptable reconstruction in terms of the location, as well as the conductivity magnitude, of the inclusions, and compared favorably with those obtained with the conventional approach. Zhou et al. [35] compared the performance of TV algorithms, such as the PDIPM, linearized alternating direction method of multipliers (LADMM), and spilled Bregman (SB) method; their experimental results showed that LADMM had the fastest calculation speed but the worst resolution, due to the exclusion of the second-derivative. PDIPM showed the sharpest change in conductivity on reconstruction, but had lower contrast than SB. SB had a faster convergence rate than PDIPM and the fewest imaging errors. Yang and colleagues [36, 37] proposed a reconstruction algorithm using enhanced adaptive group sparsity with a TV constraint, which demonstrated superior spatial resolution and better noise reduction performance than state-of-the-art algorithms, such as L1 regularization, TV regularization, and their former work on adaptive group sparsity.

With the development of artificial intelligence, deep learning has recently been applied to EIT imaging. Real-time EIT imaging with deep neural networks provides a powerful framework for post-processing convolved direct reconstructions [38]. Additionally, the dominant-current deep learning scheme for EIT is capable of fast, stable, and high-quality EIT imaging, which shows promise in terms of providing quantitative images for potential clinical applications [39]. A two-stage deep learning method has also been proposed, which may achieve highly accurate shape reconstructions, and is robust against measurement noise and modeling errors [40].

## System composition

**Fig. 1:**
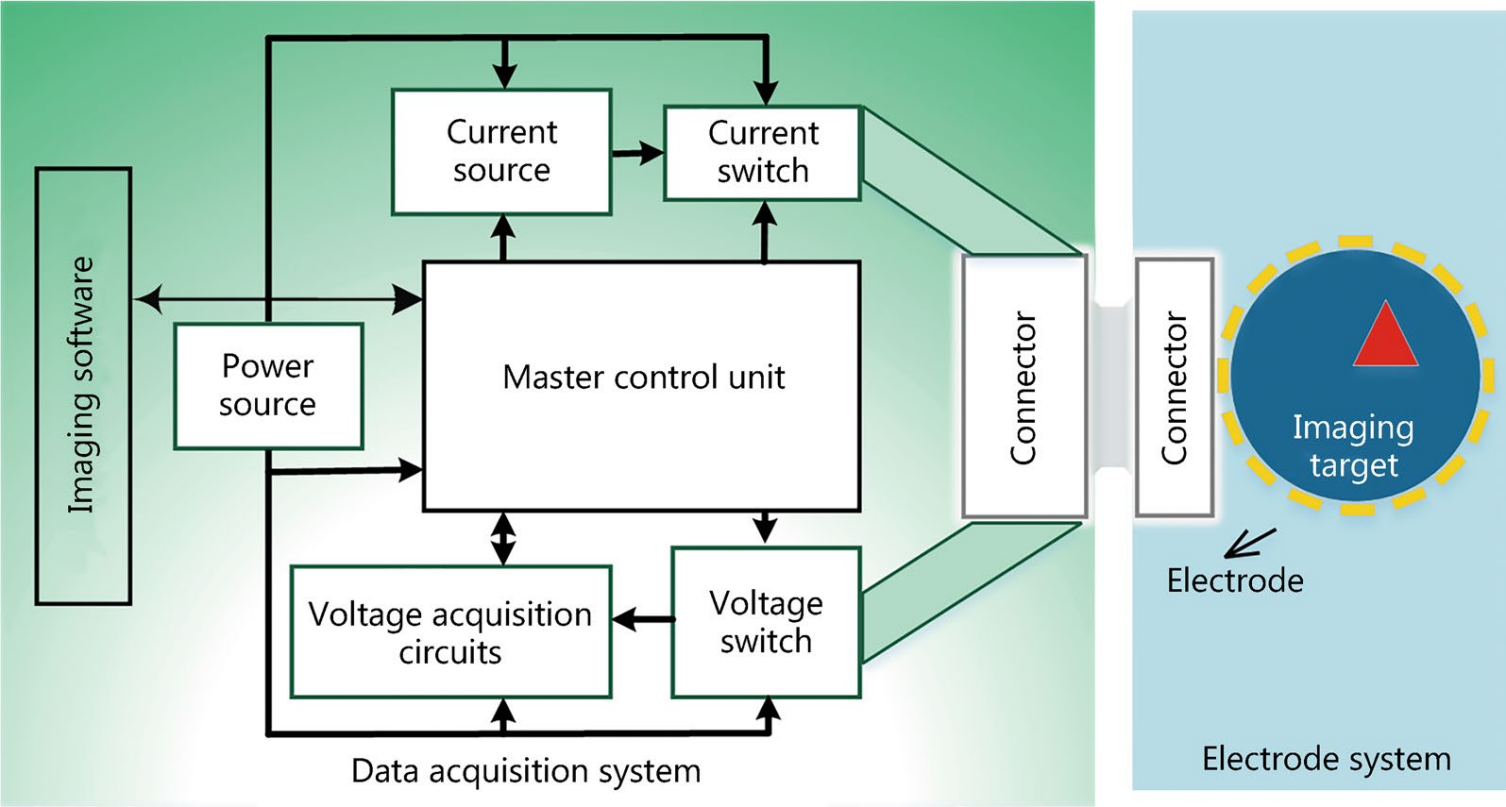
Typical composition of electrical impedance tomography (EIT) systems

The main components required to ensure accurate and stable signal acquisition in an EIT system are the data acquisition technology and electrode systems. Figure 1 depicts the typical composition of an EIT system. Based on different application requirements, data acquisition systems predominantly comprise single-channel excitation, single-channel measurement; single-channel excitation, multi-channel measurement; and multi-channel excitation, multi-channel measurement. Representative data collection systems include the OXBACT system developed at Oxford Brookes University in the UK, the ACT system developed at the Rensselaer Institute of Technology in the United States, simultaneous EIT and electroencephalography (EEG) recording system developed at the University College of London (UCL) [41–43]. These systems are suitable for laboratory research, but have yet to be validated in real-world settings.

Electrodes constitute a key component for the collection of electrical impedance information; however, there is a paucity of research regarding the influence of electrodes. In the 1990s, McAdams from UK and Jossinet from France established an electrode–skin contact model, and initially analyzed the influence of contact impedance, electrode materials, surface morphology, conductive glue, and skin preparation on electrode–skin contact impedance and measurement results [41, 42, 44].

## Applications in brain imaging

Research on craniocerebral electrical impedance imaging is critical given the lethality of brain diseases and their significant impact on the patient’s quality of life. EIT enables the recording of resistance changes that occur when ion channels open during the depolarization of neurons [45]. Because the blood has a lower impedance than the brain, impedance in the human cortex changes due to changes in cerebral blood volume during functional activity or cell swelling in patients with epilepsy [46]. For example, increased blood volume and flow in the motor cortex during motor activity would decrease the impedance; these changes, if large enough, could be imaged by EIT. A previous study reported whole-brain impedance changes after 120 min of infarction of 20.4% in rats with middle cerebral artery occlusion and − 0.1% in sham-operated rats [21]. Additionally, averaged dZ responses to ictal spike-and-wave discharges (SWDs) were characterized by a consistent impedance decrease of (− 0.31 ± 0.06) %, lasting ~ 20 ms [21]. These data suggest that EIT provides a reproducible and artifact-free means for long-term recording of impedance changes during neuronal activity [47].

EIT has exhibited broad prospects for electrophysiological measurements, diagnosis, and real-time monitoring in patients with brain diseases. Nevertheless, factors such as poor conductivity of the skull, electrode–skin contact impedance, and low precision limit the application of brain electrical impedance imaging in clinical diagnosis and treatment. This has raised challenges for the investigation of biological electrical impedance in functional brain imaging. In the subsequent section, we review the research progress in EIT in the following domains: epilepsy, stroke, brain injuries and edema, and other brain diseases.

### Epilepsy

Epilepsy is a chronic neurological disorder with a prevalence of 5–7%. At present, EEG is the main auxiliary method for the diagnosis of epilepsy. Nevertheless, EEG cannot detect epilepsy in all patients [48]. To address this issue, a parallel EIT system can be combined with intracranial EEG to improve the diagnostic yield in patients with epilepsy.

The David Holder group first employed EIT for functional neural activity imaging, successfully localizing epileptic foci using EIT with subdural electrodes [49]. Subdural electrodes are routinely used before surgery and do not significantly increase the risk to patients. Therefore, EIT could provide safer and more accurate localization of epileptic foci than intracranial EEG. Additionally, morbidity could be reduced because the need for deep penetrating electrodes is eliminated, which causes damage predominantly due to neuronal cell swelling. Intense metabolic activity results in the cell becoming unable to maintain its low internal concentration of sodium, which results in water entering the cell due to reduced intracellular osmotic potential. The extracellular space is reduced in size, and resistivity is correspondingly increased. It should therefore be possible to localize the focus of the intense activity with EIT. Cortical spreading depression can be localized using EIT with an accuracy of 8.7% of the electrode array diameter; thus, supporting the justification of trials on human patients [50].

Due to the invasive nature of subdural electrodes, Fabrizi et al. [51] subsequently employed scalp electrodes for the non-invasive localization of epileptic foci. A reliable method for defining the pre-seizure baseline and recording impedance data and EIT images was developed, in which EIT and EEG could be acquired simultaneously after filtering EIT artifacts from the EEG signal. In another study, impedance changes ranging 0.5–1.0% were observed in the raw voltage data during visually evoked responses in healthy volunteers. However, expected impedance changes of approximately 0.1% at the scalp were confounded by large movements and systematic artifacts. To remove artifacts, a method of low-pass analog filtering for EEG channels (− 6 dB at 48 Hz) and high-pass filtering for EIT channels (− 3 dB at 72 Hz), proposed by the UCL group, enabled the reduction of artifacts from 2–3 mV to 50–300 μV, thus enabling simultaneous EIT and EEG [52]. Low-pass filters were placed between the EEG recording system and the subject to filter out the high-frequency noise generated by the EIT measuring current. As low frequencies do not contain useful information for EIT, high-pass filters can be introduced between the EIT recording system and the subject to filter out low-frequency components of the artifact related to switching the applied current between electrodes. The successful reduction of artifacts has facilitated the use of EIT as an imaging technique with EEG for telemetry units, without interference by routine EEG reporting.

In 2021, EIT was used in a large, folded brain with human intracranial electrodes during ictal events for the first time [53]. EIT generated repetitive images of ictal events with the similar time course as that with functional MRI, but without its significant limitations. EIT was recorded using a system consisting of 32 parallel current sources and 64 voltage recorders. Seizures triggered by the intracranial injection of benzylpenicillin (BPN) in five pigs caused a repetitive peak impedance increase of (3.4 ± 1.5) mV (9.53%; *n* = 205 seizures); this impedance signal change was seen after only a single, initial seizure. EIT enabled the reconstruction of the seizure onset at (9.0 ± 1.5) mm from the BPN cannula and (7.5 ± 1.1) mm from the closest stereo-EEG (SEEG) contact (*P* < 0.05, *n* = 37 focal seizures in three pigs) and addressed the problems of sampling error in intracranial EEG. The amplitude of the impedance change was correlated with the spread of the seizure on SEEG (*P* < 0.001, *n* = 37). Seizures induced with procaine BPN produced cyclic epileptic activity that could be easily followed and distinguished on electrocorticogram (ECoG)/SEEG. A parallel EIT system provided a reliable method for the accurate localization of the onset of the focal seizures compared to that with ECoG/SEEG. Thus, EIT offers exciting potential in following the spread of a seizure in the near real-time and may improve the localization of seizure foci in clinical patients. The most important advantage of EIT is that it can already be used in parallel with intracranial EEG, while recording all ictal and interictal activity. EIT can produce functional images showing ictal spread with a temporal resolution of seconds, and with a spatial accuracy of less than one centimeter from the onset area, defined either by the tip of the cannula or the active SEEG electrodes. These results suggest that a combined parallel EIT system with intracranial EEG monitoring has the potential to improve the diagnostic yield in patients with epilepsy and become a vital tool in improving our understanding of epilepsy.

In 2016, the UCL group reported on EIT imaging of conductivity changes in rat somatosensory cerebral cortex, with resolutions of 2 ms and < 200 μm, during evoked potentials using an epicortical electrode array [54]. Images were validated using local field potential (LFP) recordings and current source-sink density analysis. The results demonstrated that EIT could image neural activity in a 7 × 5 × 2 mm^3^ volume in the somatosensory cerebral cortex, with greater resolution, less invasiveness, and smaller imaging volume than other methods. Subsequently, the UCL group characterized and imaged cortical impedance changes during interictal and ictal activity in the anesthetized rat, and showed that EIT could reveal the propagation of pathological activity in the cortex [48]. In this study, cortical impedance was recorded simultaneously with ECoG using a 30-contact electrode mat placed on the exposed cortex of anesthetized rats, and interictal spikes (IISs) and seizures were induced by a cortical injection of 4-aminopyridine, picrotoxin, or penicillin. A fast, transient drop-in impedance occurred as early as 12 ms before the IISs, followed by a steep rise in impedance within ~ 120 ms of the IIS. EIT images of these impedance changes showed that they were co-localized and centered at a cortical depth of 1 mm, and that they closely followed the activity propagation observed in the surface ECoG signals. The fast, pre-IIS impedance drop most likely reflected synchronized depolarization in a localized network of neurons, and the post-IIS impedance increase reflected the subsequent shrinkage the of extracellular space caused by the intense activity. EIT also captured a steady rise in tissue impedance during seizure activity, which has been previously described. Thus, EIT can detect and localize physiological changes during interictal and ictal activity, and, in conjunction with ECoG, may improve the localization of seizure foci in clinical settings in the future. To achieve this goal, the UCL group has developed and is testing a new EIT system with parallel current sources for recording impedance simultaneously over multiple frequencies, capable of implementing robust single-shot imaging of individual epileptiform events.

Based on previous research on imaging evoked activity during whisker displacement in the cortex of the anesthetized rat (with resolutions of 2 ms and < 200 μm), the UCL group went on to carry out systematic research in extending the depth of epilepsy imaging and localization. In 2018, Hannan and colleagues utilized a similar experimental approach as that in the previous paragraph to describe the trajectory of intracortical impedance changes during ictal SWDs, induced by electrical stimulation in an acute rat model of epilepsy, throughout the cerebral cortex [55]. These findings extended the knowledge of SWD initiation and expression, and demonstrated that EIT could be employed for imaging of the fast electrical activity associated with epileptic discharges, at a resolution of 300 μm and ≤ 2 ms, in the rat cerebral cortex using non-penetrating surface electrodes. These results further suggested EIT as a valuable neuroimaging tool that could improve the understanding of the neural circuits involved in the phenomenon of epilepsy.

**Fig. 2:**
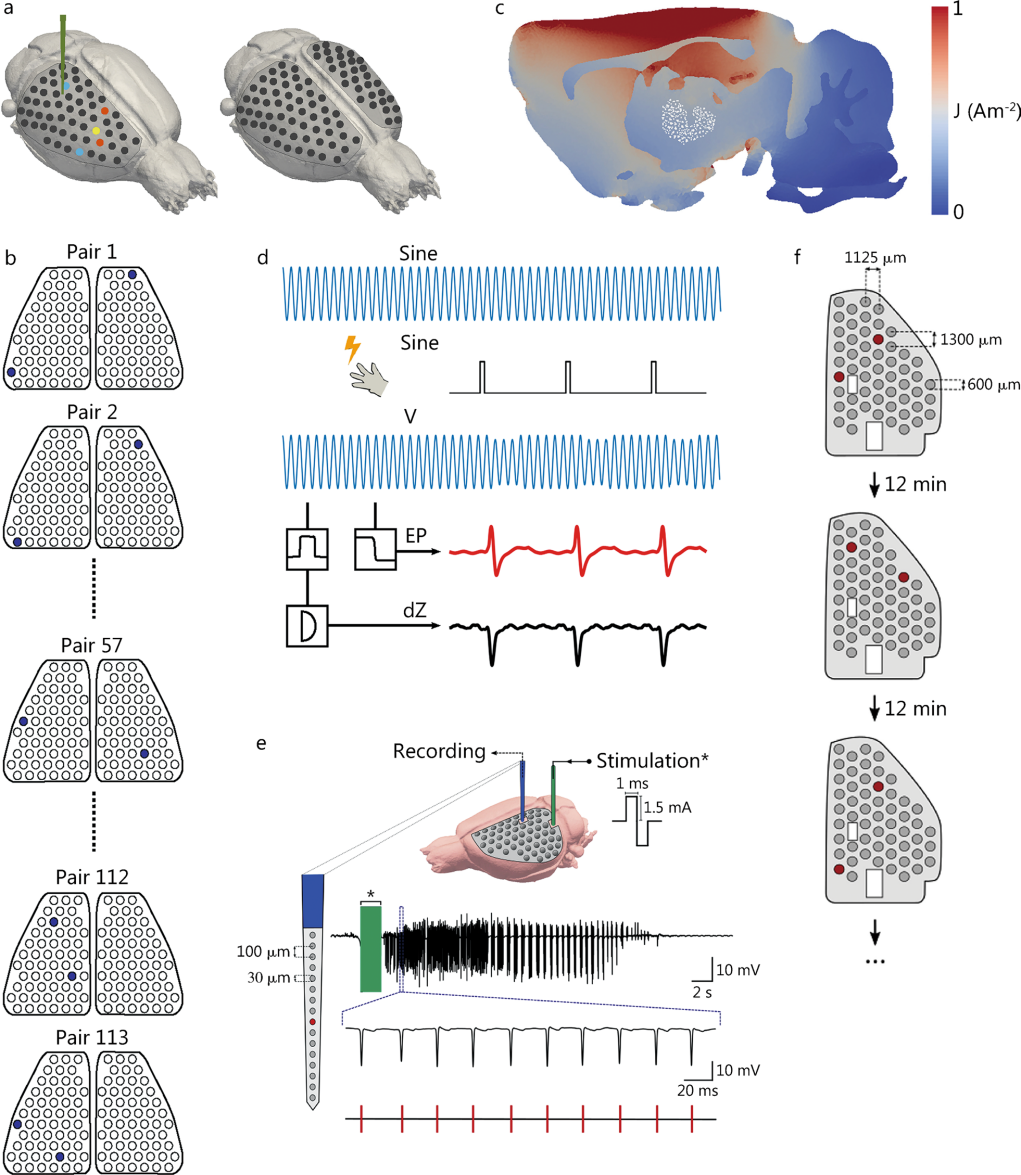
Progress in EIT for epilepsy. **a** Impedance response in the cortex and thalamus was characterized during forepaw stimulation using a 57-channel electrode array placed on the cortex and a depth electrode placed in the VPL nucleus in the thalamus. EIT imaging was conducted in an attempt to image ascending neural activity from the thalamus to the cortex using epicortical electrodes. **b** Protocol that maximizes current density in the VPL was used for imaging experiments. **c** Average current density across injection pairs concentrated in a sagittal slice when using the protocol that maximizes current density in the VPL. The location of the VPL is highlighted in white. **d** Data collection and processing work flow. **e** Angular bundle of the perforant path was electrically stimulated with a 2-s train of 100 Hz biphasic square-wave pulses to induce seizures. Pulses were 1 ms in duration per phase and 1.5 mA in amplitude. **f** During each imaging protocol, transfer impedances were recorded by injecting current through a different electrode pair on the 54-electrode epicortical array for each of ≥ 30 seizures; locations of and the distance between current-injecting electrodes were varied to ensure adequate sampling of the cortex and hippocampus. A 12-min rest period between stimulation series ensured that seizures remained stable during imaging protocols. Adapted from [56, 57]. EIT electrical impedance tomography, VPL ventral posterolateral

Furthermore, investigating the temporal characteristics of thalamic activation with respect to the imaged propagation pattern of ictal SWDs using EIT with epicortical and depth electrodes is important. Faulkner and colleagues extended the use of EIT to imaging not only from the cortex but also from deeper structures that are active in somatosensory processing, specifically the ventral posterolateral (VPL) nucleus of the thalamus [56]. For imaging purposes, two 57-channel epicortical electrode arrays were used, positioned at each hemisphere of the rat brain. The reconstructed activity was constrained to the cortical somatosensory forepaw region and no significant activity at depths greater than 1.6 mm from the surface of the cortex could be recorded. An evaluation of the depth sensitivity of EIT was investigated in simulations using estimates of the conductivity changes and noise levels derived from experiments. These indicated that EIT imaging with epicortical electrodes was limited to activity occurring 2.5 mm below the surface of the cortex (Fig. 2a–d). This depth includes the hippocampus; thus, EIT has potential in imaging activity, such as that in epilepsy, originating from this structure. To image deeper activity, however, alternative methods, such as the additional implementation of depth electrodes, are required to gain the necessary depth resolution.

In a further study, the UCL group evaluated the feasibility of using EIT to image epileptiform activity in the rat hippocampus using non-penetrating electrodes implanted on the cortical surface [57]. This experiment revealed a focus of neural activity localized to the dentate gyrus, which was spatially and temporally aligned to LFP recordings, and could be reproducibly reconstructed in all animals, with a localization accuracy of ≤ 400 μm (*P* < 0.03125, *n* = 5). These findings represent the first experimental evidence of the ability of EIT to image neural activity in subcortical structures from the surface of the cortex with high spatiotemporal resolution, suggesting that this method may be used for improving the understanding of functional connectivity between cortico-hippocampal networks in both physiological and pathophysiological states. The depth sensitivity of EIT for imaging epileptiform neural activity using epicortical electrodes has, therefore, been shown to extend to the hippocampus, corresponding to a penetration depth of at least 3 mm (Fig. 2 e, f). Furthermore, the UCL group is currently investigating the feasibility of employing electrical imaging activity for deeper structures, such as the thalamus, using similar approaches.

To optimize EIT for measuring the activity in cortical epilepsy, electrical stimulation of the cerebral cortex was employed to induce seizures in anesthetized rats, thereby enabling the characterization of the frequency of responses with fast and slow impedance changes during epileptiform activity [58]. The authors concluded that the optimal frequency for imaging epileptiform activity was 1355 Hz, which maximized the signal-to-noise ratio (SNR) of fast neural changes, while enabling the simultaneous measurement of slow changes. In addition, the UCL group presented two adapted in vivo seizure models, the neocortical and hippocampal epileptic after-discharge models [59], enabling stereotyped seizures to be induced on demand by electrical stimulation in anesthetized, neurologically intact rats. The proposed models provided an efficient method for the high-throughput screening of novel anti-seizure therapies, including closed-loop stimulation paradigms, and were well-suited to in vivo investigations that required tight regulation of seizure timing under anesthetized conditions, especially for neuroimaging studies aimed at understanding the development of epileptogenic networks. Based on this proposed model, Hannan and colleagues characterized and imaged the slow impedance response during neocortical and hippocampal epileptiform events in the rat brain, and evaluated its relationship to the underlying neural activity [60]. Slow impedance responses were consistently time-matched to the end of seizures, and EIT images of this activity were reproducibly reconstructed in all animals (*P* < 0.03125, *n* = 5). These findings suggest that the slow impedance response is a reliable marker of hypersynchronous neuronal activity during epileptic seizures, and can thus be utilized for investigating the mechanisms of epileptogenesis in vivo and aiding in the localization of the epileptogenic zone during the pre-surgical evaluation of patients with refractory epilepsies.

Xiuzhen Dong's group [61] in China has focused on epilepsy prediction, treatment, and real-time imaging. This group has developed a novel method for the rapid prediction of epilepsy using nonlinear analysis of EEG data in a rat epileptic model, and demonstrated the clinical utility of a phase-synchronized analysis algorithm for predicting epileptic seizures [62]. Subsequently, they developed a responsive electrical stimulation system coupled with the phase-synchronized algorithm to predict seizures, which significantly inhibited epileptic seizures [63]. Furthermore, they successfully utilized EIT to monitor seizure progress in real-time; EIT and EEG data were recorded simultaneously in rats, indicating that EIT can be used to detect and image electrical impedance reduction within lesions during epileptic seizures [64].

Future work should focus on exploiting the advantages of EIT as an imaging tool, including its ability to image neural activity at the mesoscopic scale over large volumes and to delineate the spatiotemporal dynamics of long-range cortical-hippocampal networks in both physiological and pathological states.

### Stroke

Stroke is the acute onset of a cerebrovascular event that can be classified into two clinical types: ischemic stroke, which includes transient ischemic attacks, intracranial embolisms, and thrombosis; and hemorrhagic stroke, which includes cerebral hemorrhage and subarachnoid hemorrhage [65]. Different types of stroke vary in terms of treatment approaches and urgency. Hence, neuroimaging techniques that enable early identification and rapid detection are necessary. However, current imaging modalities, such as computed tomography (CT), MRI, and cerebrovascular angiography, are unable to fully meet these requirements. A combination of CT, angio-CT, and CT perfusion is very useful and efficient in clinical stroke imaging. Due to the lack of a rapid and accurate diagnosis on neuroimaging scans, patients with stroke often fail to receive timely treatment [66]. Differences in the conductivity spectrum of normal, hemorrhagic, and ischemic cortical tissue have been reported [67]. A seminal study on stroke in anesthetized rats revealed that cerebral ischemia caused an increase in impedance of up to 60%, and cortical electrodes detected an increase in impedance of 10–20% [68]. The fast and portable features of EIT are expected to facilitate the early diagnosis and identification of stroke to ensure that patients receive timely treatment.

David Holder’ group [69] assessed the utility of multi-frequency electrical impedance tomography (MFEIT) in the imaging, diagnosis, and differentiation of hemorrhagic and ischemic strokes. This group has reported on the most suitable pattern for stroke imaging, as well as the feasibility of using MFEIT for the early diagnosis and imaging of thrombolysis and stroke in a head model with real electrical conductivity [70, 71]. They employed frequency-difference EIT imaging to identify hemorrhagic and ischemic strokes [72], and demonstrated that time-difference images were less feasible for ischemic stroke than for hemorrhagic stroke [13]. Jehl et al. [73] have also reported on hardware systems and imaging algorithms and indicated that a generic head mesh is sufficient for monitoring patients for secondary strokes following head trauma, thereby precluding the need for patient-specific head meshes for image reconstruction. They utilized the Jacobian matrix with electrode movement to correct the electrode model in MFEIT and improve the imaging quality [74]. In 2018, David Holder’s group, in collaboration with the Hyper Acute Stroke Unit (HASU) at UCL Hospital (UCLH), conducted a clinical trial that collected MFEIT data from 23 stroke patients and ten healthy volunteers. This dataset was combined with EEG, CT, and MRI data in stroke patients to form the basis of a stroke classification scheme [75].

**Fig. 3:**
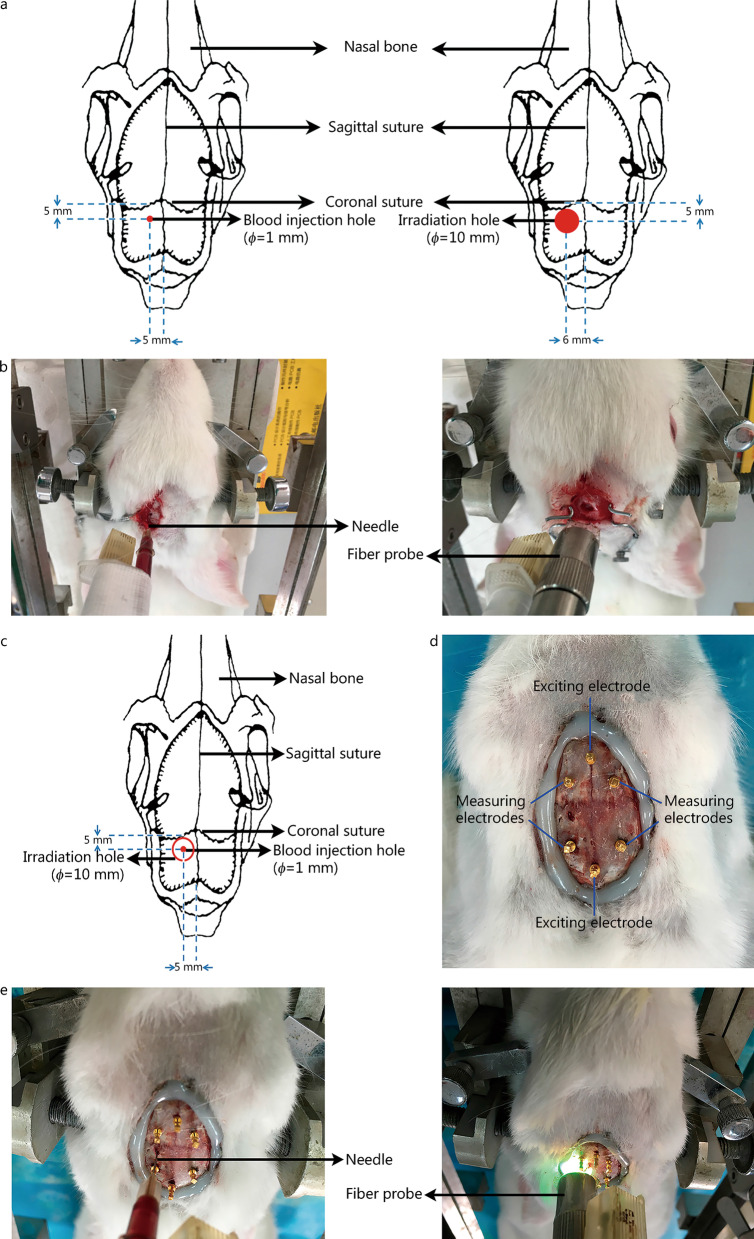
Comparison of ex vivo and in vivo characterization of bioimpedance spectroscopy of normal, ischemic, and hemorrhagic brain tissue at frequencies of 10 Hz–1 MHz in rabbits. **a, b** Hemorrhagic model obtained with the autologous blood injection method and the ischemic model obtained with the photothrombotic method. **c** Schematic diagram. **d** Electrode distribution for in vivo measurement of whole-brain impedance spectra. **e** Intracerebral hemorrhage model (autologous blood injection method) and ischemic model (photochemical induction method). Adapted from [86, 87]

Unlike the UCL team, Xiuzhen Dong’s group [27, 76–81] predominantly has focused on the real-time monitoring of brain diseases using EIT, and has generated preliminary products in image monitoring, with phased results. In one study, in vivo measurements of rabbit brain tissue impedance were obtained under both normal and ischemic conditions using dual-electrode measurements in the frequency range of 0.1 Hz–1 MHz. The results indicated that functional brain changes caused by a local deficiency in blood could be detected and imaged using EIT [82]. Another study demonstrated that EIT could enable the rapid and sensitive detection of potential cerebral ischemia [83]. Furthermore, an exploratory study successfully reconstructed the impedance images of unilateral stroke lesions using symmetrical EIT [84]. Real-time imaging of focal cerebral infarction in rabbits using EIT has also been reported [85]. The Fourth Military Medical University (FMMU) group successively measured and analyzed the impedance spectroscopy of normal, hemorrhagic, and ischemic brain tissue within 10 Hz–1 MHz using ex vivo and in vivo approaches in rabbits for stroke identification (Fig. 3). Their findings indicated that impedance spectra could be used to distinguish normal, ischemic, and hemorrhagic brain tissue types, supporting the use of MFEIT to differentiate stroke types [86, 87].

The FMMU group has performed pioneering studies on hemorrhagic stroke. Dai and colleagues [76] used in vivo imaging of spiral drilling and drainage for the treatment of subdural hematoma, monitoring the condition of patients with intracranial hemorrhage (ICH), and detecting real-time changes in human intracranial resistivity during surgery. These achievements highlight the potential of EIT as a routine neuroimaging tool for monitoring ICH. Most patients with ICH require a craniotomy. Dai and colleagues [88] investigated the effects of an open or closed cranium on impedance changes in the brain in a rabbit ICH model and reported that cranium completeness (open or closed) influenced impedance changes within the brain when using EIT to monitor ICH. Therefore, cranium completeness should be considered in future studies when applying EIT to monitor cerebral hemorrhage.

Subarachnoid hemorrhage is one of the most severe emergencies in neurosurgery. Xiuzhen Dong’s group [80, 89] achieved real-time detection and continuous monitoring of subsequent pathological states by establishing subarachnoid hemorrhage and ICH models in piglets. Other groups have conducted studies on the detection of small bleeding in the brain, intraventricular hemorrhage, and the use of support vector machine classifiers for detecting cerebral hemorrhage [90–92].

### Brain injuries and brain edema

Cerebral edema is a major risk factor for high morbidity and mortality, underscoring common neurological diseases. Clinically, there is an urgent need for a diagnostic tool that enables real-time monitoring of brain edema and the distinction of brain edema types. Due to the electrical impedance difference between normal cortical tissue and brain edema tissue, EIT has potential in the detection of brain edema.

**Fig. 4:**
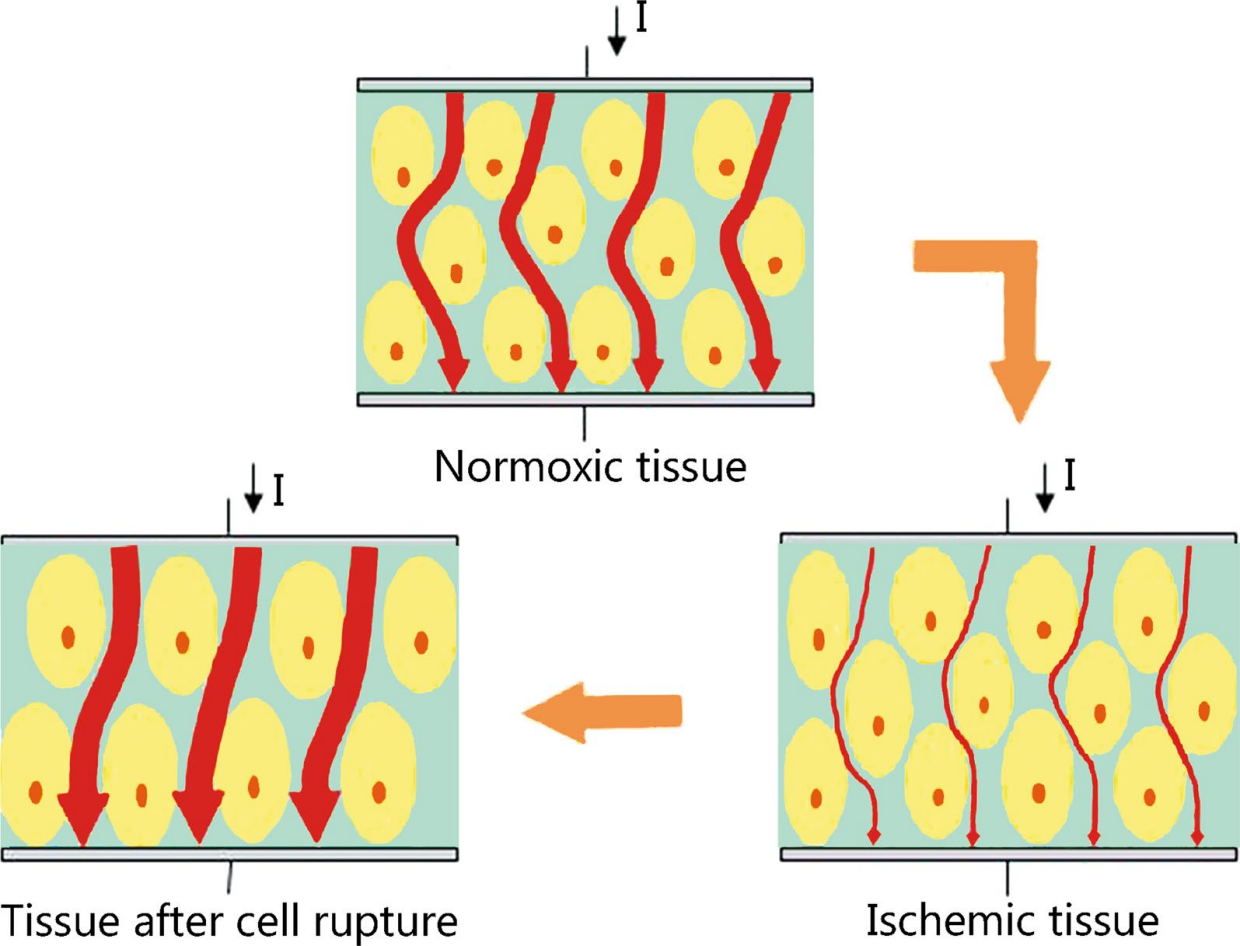
Brain tissue changes at different phases of cerebral edema. At an early stage of cerebral edema, cellular expansion reduces the intercellular space which decreases electrical current density through tissue under the same excitation, thus increasing tissue impedance. Subsequently, necrotic or ruptured neurocytes enhance cellular membrane permeability. At this stage, electrical current easily passes through brain tissue, which results in a further reduction in tissue impedance. Adapted from [96]

Fu et al. [93] reported on the use of EIT for real-time and non-invasive monitoring of regional cerebral edema during a clinical dehydration treatment. Further, assessments of current reconstruction algorithms for the continuous monitoring of brain injuries have been conducted [94, 95]. Cerebral edema can be secondary to ischemic brain injury. In 2018, Song et al. [96] measured variations in electrical impedance at different phases of cerebral edema in rats with ischemic brain injury and analyzed the corresponding morphologic changes (Fig. 4). Their results demarcated the feasibility of using EIT to monitor cerebral edema in real-time and to distinguish between different types of brain edema. The progression of brain edema is closely associated with brain water content, which is reflected in the intracranial pressure. The FMMU group reported on the feasibility of using EIT to monitor changes in the brain water content related to brain edema and highlighted its utility as a non-invasive imaging tool for the early recognition of cerebral edema and evaluation of mannitol dehydration [14].

Other research groups have investigated the translatability and clinical applicability of EIT. For example, Lan et al. [97] examined the application of brain edema monitors based on EIT in infant cardiopulmonary bypass, and Manwaring et al. [98] employed a novel intracranial pressure/EIT electrode combination sensor bedside system to monitor and manage patients with brain injuries.

### Other applications of EIT

EIT has clinical application prospects for brain diseases such as cerebral hematomas, brain abscesses, and brain neoplasms. Table 2 summarizes the brain disease impedance spectra for various diseases and the model systems used. Tian et al. [99] employed EIT for the real-time monitoring of cerebral hematoma. A standard ring full array and semi-array EIT were used to detect, locate, and quantify simulated intracranial hematoma in an in vitro ovine model, providing data for the early diagnosis of cerebral hematoma [100]. Kim et al. [101, 102] performed in vivo experiments in a brain abscess model to validate the MREIT technique; they reported conductivity information of tissues in situ for clinical applications, and successfully used a dual-frequency range conductivity mapping, magnetic resonance method to monitor dynamic changes in the state of brain abscesses. Benabid et al. [103] examined the correlation between brain tumor tissue structure and corresponding impedance values, whereas Bullard et al. [104] combined data on the changes in brain impedance characteristics with the corresponding CT density. Data analysis revealed that a decrease in impedance typically corresponded to low-density regions; conversely, an increase in impedance corresponded to enhanced lesions [104]. Other groups have attempted to use MREIT to detect and image brain tumors [105, 106]. In addition to its diagnostic value, EIT enables the real-time monitoring of brain function during surgery [107, 108].

**Table 2:** Brain disease impedance spectra

Disease	Model/species	Trends of impedance changes
Epilepsy	In vivo/rat	Impedance decreased gradually during a seizure and reached a minimum at the end of the seizure. Following seizure activity, the impedance returned to the interictal baseline or increased to a level above the baseline
Stroke	Ex vivo/rabbit In vivo/rabbit	Impedance spectra of stroke lesions significantly differed to those of normal brain tissue; the ratio of change in impedance of ischemic and hemorrhagic tissue with regard to frequency was distinct; tissue type could be distinguished according to impedance spectra
Brain injuries and cerebral edema	23 patients with brain edema	Overall impedance across the brain increased significantly before and after mannitol dehydration treatment (*P* = 0.0027)
	Ex vivo/male rats	After the first 6 h following the onset of ischemic brain injury, the resistivity of brain tissue increased (*P* < 0.05); from 6 to 24 h, the resistivity of brain tissue decreased
	Patients with cerebral hemorrhage	Dehydration effects induced changes in average reconstructed impedance value and intracranial pressure exhibited a strong negative correlation in all patients (mean correlation: *R*^2^ = 0.78 ± 0.16, *P* < 0.001)
Brain abscess	In vivo canine model	Relative conductivity contrast ratios (rCCR, %) of central abscess lesions were higher than those of surrounding areas at 6, 12, and 18 h (*P* < 0.01). Over 12 h, the relationship between induction time and rCCR exhibited a positive correlation followed by a negative correlation (*P* < 0.01)
Brain neoplasms	Three-dimensional finite element model	Tumor-like anomalies with 200% conductivity contrast were straightforwardly detected and imaged using an existing 3 T system with total acquisition time under 30 min

## Limitations

Table 3 lists the research progress in brain electrical impedance tomography. At present, four key factors still limit the application of EIT in brain imaging: (1) low skull conductivity and electrode–skin contact impedance; (2) modeling accuracy; (3) reconstruction algorithms; and (4) drive pattern and current density protocols.

**Table 3:** Research progress in brain electrical impedance tomography

Disease	Research group	Method	Research results
Epilepsy	Holder D	EIT with subdural electrodes	Localization of epileptic foci [49]
		Combining EEG telemetry and EIT data	EIT detected and localized different physiological changes during interictal and ictal activity [47] Changes in EIT were consistent with electrogram activity during seizures [51]
		Non-penetrating surface electrodes	Cortical EIT epilepsy imaging [55]
			Deeper neural activity Imaging, penetration depth ≤ 2.5 mm below the cortex [56]
			Hippocampus imaging, penetration depth ≥ 3 mm below the cortex [57] Optimization of cortical EIT epilepsy imaging [58]
	Dong X	Nonlinear dynamic methods	Seizure prediction [59, 60]
		Responsive electrical stimulation system	Epilepsy prediction and seizure suppression [63]
		EIT	Real-time imaging of epileptic seizures [64]
Stroke	Holder D	MFEIT	Imaging and differentiation of hemorrhagic and ischemic stroke [69]
		Jacobian matrix	Improved imaging quality [74]
		Analysis of MFEIT, EEG, CT, and MRI data	Basis of future research into stroke classification [75]
	Dong X	MFEIT	Detection and imaging of cerebral ischemia [82, 83]
			Impedance spectroscopy of normal brain tissue and hemorrhagic and ischemic stroke injury [86]
			Differentiation of normal, ischemic, and hemorrhagic brain tissue types based on impedance spectroscopy [87]
		Twist drill drainage for subdural hematoma	Intraoperative real-time monitoring and measurement of intracranial hemorrhage [76]
Brain injuries and brain edema	Dong X	EIT	Real-time and noninvasive monitoring of local brain edema [93]
		Dynamic EIT	Evaluation and trial of performance of several different EIT algorithms in continuous monitoring of brain injury [94, 95]
		1260 Impedance/Gain-Phase Analyzer	Measurement of electrical impedance at different stages in a rat model of brain edema after ischemic brain injury [96]
			Real-time monitoring and differentiation of brain edema [14]
		16-electrode EIT system	Changes in brain water content associated with cerebral edema and monitoring of intracranial pressure and brain impedance imaging [14]
Brain abscess	Kim HJ	MREIT	Comparative information on new brain abscess lesions [101]
			Characterization of time course changes before and after brain abscess induction [102]
Brain neoplasms	Farnarier P	Stereoimpedoencephalography (SIEG)	Relationship between brain tumor tissue impedance and normal tissue impedance [103]
	Bullard DE	Monopolar and bipolar impedance monitoring	Combination of changes in brain impedance characteristics with corresponding CT density [104]
	Kim HJ Muftuler LT	MREIT	Feasibility of MREIT conductivity imaging for brain tumor detection [105, 106]

*EIT* electrical impedance tomography, *MFEIT* multifrequency electrical impedance tomography, *MREIT* magnetic resonance electrical impedance tomography, *EEG* electroencephalography, *CT* computed tomography, *MRI* magnetic resonance imaging, *PET* positron emission tomography

### Skull conductivity and electrode–skin contact impedance

Low skull conductivity and electrode–skin contact impedance raise some practical challenges to EIT brain imaging. Jiang et al. [109] studied the application of capacitively coupled electrical impedance tomography (CCEIT) in head imaging. CCEIT is a non-contact EIT technique that uses voltage excitation without direct contact with the skin, unlike in EIT, where the current is directly injected into the skin. Practical experiments were carried out with a 12-electrode CCEIT phantom, saline, and carrot samples. Experimental results showed the potential and feasibility of CCEIT for stroke imaging. In addition, a previous study suggested that EIT is relatively insensitive to variations in neonatal skull impedance [110]. In 2011, Mccann et al. [111] reported on a new system called fEITER. The system was designed and built to use EIT to enable functional imaging of the human brain by integrating scalp electrodes with evoked response stimulation, with a good SNR. To enhance the contrast in brain imaging, Nissinen et al. [112] proposed an approach based on the Bayesian approximation error method. In this approach, both the geometry and skull conductivity are embedded in the approximation error statistics, contributing to a computationally efficient algorithm that can significantly improve the specificity and sensitivity in detecting the characteristics of internal hemorrhage.

The long-term stability and composite impedance of the electrode-tissue system depend largely on the type of electrodes used [113]. Xu et al. [81] compared five common types of Ag/AgCl bio-electrodes in terms of contact impedance, SNR, uniformity, and stability, based on impedance data from 10 healthy adult volunteers, obtained using 2-electrode and 16-electrode methods. The results showed that the Ag/AgCl powder electrode had low contact impedance, high SNR, better uniformity, and greater stability, suggesting that it was likely to become the optimal choice for brain EIT measurements, providing feasible technical support for further application in cerebral EIT. Yang et al. [114] subsequently demonstrated that the combination of Ag/AgCl powder electrode and low viscosity conductive gel may be the best choice for brain EIT. Compared to the scalp electrodes, subdural voltage sensing electrodes are more sensitive but allow lower current [115]. In epilepsy imaging, electrodes may not be stable when placed on the head. By filtering out abnormal coefficients with discrete wavelet transforms, multiple disconnected electrodes can be detected and invalid data frames after disconnection can be compensated [116]. This research has improved the clinical applicability of dynamic brain EIT, contributing to its further promotion. Furthermore, a piecewise processing method (PPM) has been introduced to manage erroneous EIT data after the reattachment of faulty electrodes [117]. Without iterative calculations, the PPM can efficiently manage erroneous EIT data after the reattachment of faulty electrodes.

### Modeling accuracy

Heterogenic and anisotropic electric properties of human tissues make accurate modeling and simulation very challenging, leading to a tradeoff between physical accuracy and technical feasibility. De Marco et al. [118] proposed a complete algorithm flow for an accurate EIT modeling environment featuring high anatomical fidelity with a spatial resolution equal to that provided by MRI and a novel realistic, complete electrode model implementation. They demonstrated that current graphics processing unit (GPU)-based platforms provide sufficient computational power for a domain discretized with five million voxels to be numerically modeled in about 30 s.

Structural similarity of a head model can affect the accuracy of the forward solution to EIT. Generally, the head model is a four-concentric circle model (FCCM) that ignores the inhomogeneous conductivity distribution of a real skull. To decrease the errors caused by using FCCM, a more accurate head model, known as that inhomogeneous skull model (ISM), has been proposed, and a brain EIT reconstruction algorithm incorporating ISM has been developed [78]. Simulation results have shown that image quality and localization accuracy can be improved by using ISM. Additionally, the reconstructed image may be more sensitive to the location of bony sutures than to the variations in skull thickness. In conclusion, incorporating skull inhomogeneity into image reconstruction effectively improves image quality and localization accuracy for brain EIT.

In addition, the skull type, realistic anatomy, and cranium completeness affect impedance detection. the FMMU group studied the impedance models of different skull types [119], and provided accurate skull modeling to improve the accuracy of related research on bioelectricity in the head and the biological effects of the electromagnetic field. Additionally, a novel head phantom that features realistic anatomy and spatially varying skull resistivity has been designed and fabricated based on 3-dimensional printing techniques [77]. This phantom provides a standardized, efficient, and reproducible method for the construction of a head phantom for EIT that could be easily adapted to other fields in brain function research, such as transcranial direct current stimulation and EEG [120]. The FMMU group also confirmed that cranium completeness (open or closed) affects impedance changes within the brain when using EIT to monitor ICH [88]. Hence, cranium completeness should be considered when establishing an ICH model and analyzing the corresponding EIT results.

The head phantom contains three layers, representing the scalp, skull, and brain tissues. Incorporating realistic geometry and conductivity into the reconstruction algorithm significantly improves the quality of brain EIT imaging [46]. Furthermore, the addition of shells to the forward model improved image quality, as expected, with an analytical model for reconstruction [121]; however, the finite element model (FEM) method was employed, which used a medium-mesh and a linear element computation, requiring improvements to yield the expected benefits.

Body tissues, especially white matter and the skull in head imaging, are highly anisotropic. Therefore, incorporating anisotropy in numerical models used for image reconstruction may improve EIT image quality. Abascal et al. [122] developed a method for incorporating anisotropy in a forward numerical model for head EIT, and assessed the resulting improvement in image quality in the case of linear reconstruction of one example of the human head. A FEM of a real adult human head, with segments for the scalp, skull, cerebrospinal fluid (CSF), and brain was produced from a structural MRI. Anisotropy of the brain was estimated from diffusion tensor-MRI of the same subject, and anisotropy of the skull was approximated using the structural information.

Anatomically accurate (head-shaped) finite element (FE) meshes have also been shown to improve the quality of reconstructed images. A robust protocol for the rapid generation of patient-specific FE meshes from MRI or CT data can provide a practicable and rapid method for the generation of patient-specific FE meshes of the human head that are suitable for EIT [123]. The proposed protocol comprises four steps: segmentation, surface extraction, surface mesh processing, and meshing.

### Reconstruction algorithms

A suitable reconstruction algorithm is used to shorten the imaging time, improve the imaging quality and SNR, and reduce external interference, such as noise and head movement. With pre-processing, reconstruction using linear methods may take up to a few minutes per image, even for detailed meshes. However, iterative non-linear reconstruction methods require larger computational resources, and reconstruction of the images with detailed meshes consumes too much time for clinical use. To solve this timing bottleneck, the UCLH group has used the resources of the GRID middleware, “Condor”, and a cluster of 920 nodes; the reconstruction speed for EIT images of the human head with a non-linear algorithm was increased by 25–40 times that for serial processing of each image [124].

Tikhonov regularization is a commonly used reconstruction method. Its main principle is to apply damping constraints to the matrix inversion procedure in the imaging process to make the solution stable [125]. When using this method for image reconstruction, the solution will inevitably involve deviation. Therefore, it is necessary to improve Tikhonov's regularization. To this end, the UCLH group introduced a weighting scheme that normalizes the sensitivity matrix for voxels at different depths [126]. This increases the number of linearly independent components that contribute to the solution, and forces different measurement patterns to achieve similar sensitivity. When applied to stroke, this weighted regularization improved overall image quality.

Liston et al. [127] described a new reconstruction algorithm based on a forward solution that modeled the head as four concentric spherical shells, along with conductivities of the brain, CSF, skull, and scalp. The model predicted that the mean current traveling in the brain in the diametric plane for current injection from polar electrodes was 5.6 times less than that obtainable if the head was modeled as a homogeneous sphere; thus, an algorithm based on this approach should be more accurate than one based on a homogeneous sphere model [127]. Statistical parametric mapping (SPM) may also be used on EIT images, which extract improved images from clinical data series with a low SNR [128].

During prolonged dynamic brain EIT clinical monitoring, head movement interference, from patient body movements and nursing procedures performed by medical staff, is a common problem. Li et al. [94] compared the performance of different algorithms in the context of dynamic brain monitoring and concluded that a novel algorithm, known as SB-IBCD, was the most effective in unveiling small intracranial conductivity changes. Furthermore, noise and interference may cause insufficient image quality. To evaluate the performance of the signal processing-prior information-based reconstruction (SPR) method, Haoting Li and colleagues carried out simulation and in vivo experiments. The results showed that SPR could improve brain EIT image quality and recover intracranial perturbations from certain incorrect measurements [129]. In addition, the FMMU group proposed an online strategy to manage head movement interference in brain EIT data based on the distribution characteristics of wavelet coefficients [130]; the strategy reduced movement interference in the data and improved the quality of the reconstructed images.

### Drive pattern and current density protocols

To develop a single-source drive pattern more suitable for brain EIT, the FMMU group built a more realistic experimental setting that simulates the head, with the resistivity of the scalp, skull, CSF, and brain [27]. Adjacent, cross, polar, and the newly proposed pseudo-polar drive patterns were compared in terms of boundary voltages, dynamic range, and independent measurement number. The results showed that the pseudo-polar drive pattern was optimal in every aspect, except for the dynamic range. The polar and cross drive patterns came next, and the adjacent drive pattern was the least optimal.

According to the UCL group, maximizing the current density provides the best protocol for fast neural EIT applications [131]. However, prolonged cortical stimulation may induce neural injury through excitotoxicity and electrochemical reactions at the tissue-electrode interface. Hannan et al. [132] further assessed whether current levels used in fast neural EIT studies induced histologically detectable tissue damage when applied continuously to rat cerebral cortex. Histological analysis revealed that a continuous injection of 100 µA current, equating to a current density of 354 Am^−2^, into the rat cerebral cortex at 1725 Hz did not cause cortical tissue damage or any neuronal morphological alterations. Hence, the safety of current injections during typical EIT protocols for imaging fast neural activity has been validated.

In addition, to improve the SNR, a novel method, in which contemporaneous evoked potentials are subtracted, has been presented, with a current applied at 225 Hz to the cerebral cortex during evoked activity [133]. This method provides a reproducible and artifact-free means for recording resistance changes during neuronal activity, which could form the basis for imaging fast neural activity in the brain.

## Combination approaches and future directions

### MREIT

MREIT is a newly developed impedance imaging method that provides images with accurate high-resolution cross-sectional resistivity [134–136]. MREIT delivers useful information about important conductivity values in the brain and is widely used in EEG and magnetoencephalogram source location and imaging. MREIT enables the generation of high-resolution electrical conductivity images and the measurement of magnetic flux density in three-dimensional space, without being severely affected by the low conductivity of the skull. Various studies have applied MREIT in cancer imaging, assessed the feasibility of three-dimensional MREIT for detecting hemorrhagic and ischemic strokes, and employed non-invasive magnetic flux density measurements to estimate intracranial conductivity [105, 106, 137–140]. As commonly practiced in medical imaging, cross-modal approaches have become a trend in bioelectrical impedance imaging research.

### Microelectrode array EIT

Compared with non-invasive electrodes, invasive electrodes implanted in the skull provide better spatial resolution, higher SNR, wider frequency range, and are less susceptible to motion artifacts and external noise. The UCL group proposed a method for reconstructing tomographic images of fast neural activity with EIT using intracranial planar arrays. Using a 256-electrode array covering the brain, simulated deep brain hippocampal or thalamic activity could be localized, with an accuracy of 0.5 mm. Further, parallel studies achieved a temporal resolution of 2 ms for imaging fast neural activity using EIT [141]. These findings highlight the advantages of invasive electrodes. Nevertheless, traditional electrode materials have high rigidity relative to the human skull. This mismatch in mechanical properties can result in brain damage [142], which limits the application of this technology for clinical diagnosis and treatment.

**Fig. 5:**
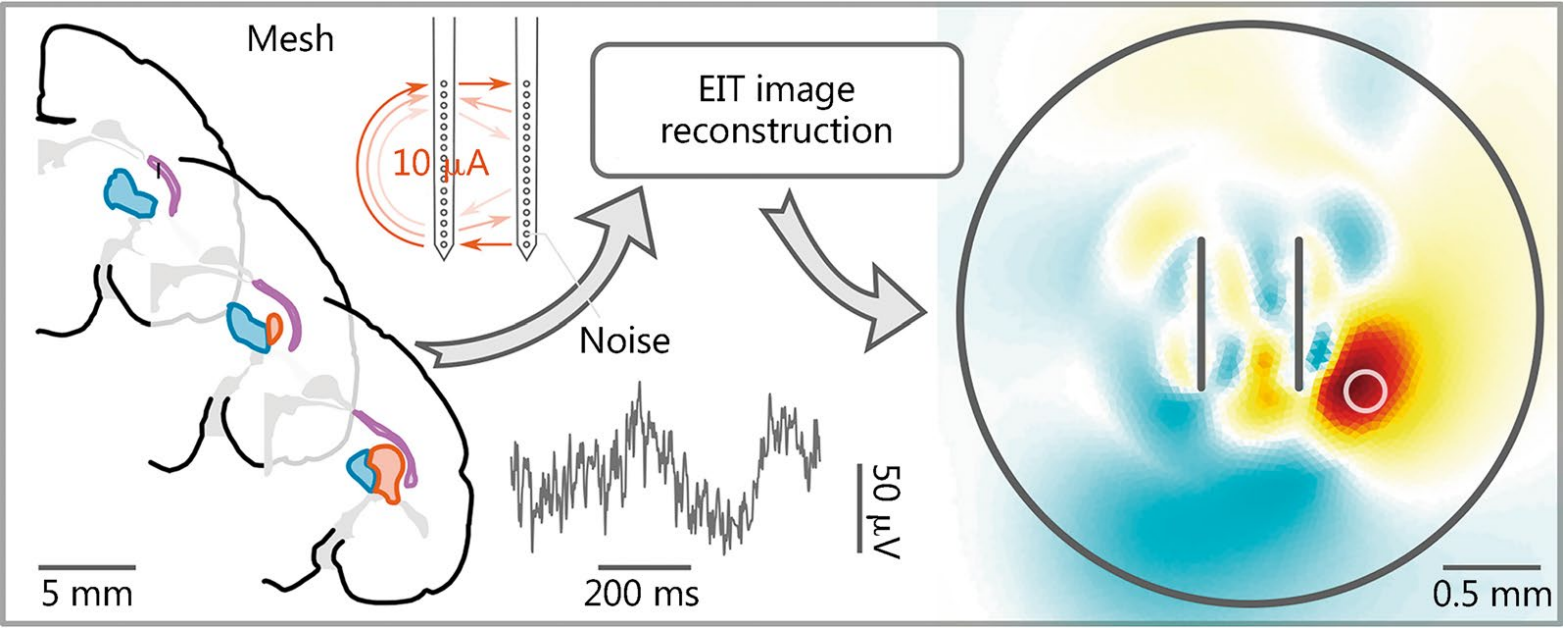
Microelectrode array EIT was simulated using an anatomically accurate marmoset brain model. Adapted from [145]. EIT electrical impedance tomography

In recent years, the development of flexible materials has provided a potential solution to safety issues associated with implanted electrodes. Electrodes composed of flexible materials have enabled safe and non-invasive in vivo brain recordings with microelectrode arrays. Natural biomaterials that have evolved over millions of years have similar elastic and shear modulus features to those of brain tissue, with good biological properties, and can adapt to the curved topology of the brain. Kim et al. [143] and Wang et al. [144] used a flexible substrate prepared from silk bioprotein to transfer polyimide mesh microelectrode arrays to cat brains. This silk protein substrate spontaneously dissolves when contacting water. When the silk protein is completely ablated, the polyimide mesh microelectrode array can be attached to the brain, thus realizing non-invasive signal measurements [143, 144]. In 2019, Zhu et al. [145] reported a microelectrode array EIT technique that enables fast functional imaging in the thalamus. This technique enables the simultaneous reconstruction of concurrent activity in multiple subcortical bodies (Fig. 5). These non-invasive approaches may promote the safety of implanted electrodes and overcome the issues of high skull resistivity, scalp leakage of the drive current, and electrode–skin contact impedance.

Microelectrode array EIT is a pioneering approach for the diagnosis and treatment of brain diseases. For instance, patients with glioma typically require craniotomy to remove tumor lesions. When the maturity and safety of the technique have been ensured, flexible microelectrodes can be placed on surgically resected tumor lesions to monitor therapeutic effects in post-surgical patients with glioma and to identify the recurrence and false progress of gliomas in real-time.

## Conclusion

After decades of development, EIT has achieved breakthrough results in brain imaging. In terms of epilepsy imaging, the EIT imaging depth has been extended from not only the cortex but also deeper structures, such as the hippocampus, in a rat model of epilepsy. Furthermore, EIT data has been collected in patients with epilepsy, providing support for multi-modal imaging. In stroke diagnosing, the FMMU group has measured the impedance spectrum of cerebral hemorrhage and cerebral ischemia in rabbits, and developed a bedside EIT stroke monitoring system for clinical practice. In addition, EIT has been applied to monitor the changes in brain water content associated with cerebral edema, which could provide a real-time and non-invasive imaging tool for the early identification of brain edema and the evaluation of mannitol dehydration.

EIT provides a novel method to improve coverage and seizure onset localization. The feasibility of EIT has been previously assessed in a computer simulation, which revealed better accuracy in seizure detection with EIT than with intracranial EEG. Additionally, the UCL group has demonstrated that combining a parallel EIT system with intracranial EEG monitoring has the potential to improve diagnostic rates for patients with epilepsy and become an important tool in improving our understanding of epilepsy. In the diagnosis of cerebrovascular diseases, continuous real-time EIT monitoring may be an effective complement to traditional imaging methods, such as CT and MRI, because it can enable early detection of intracranial pathological changes and differentiate stroke types, thus guiding physicians to perform further inspection or administer protective medicine to improve the prognosis of the patient. Regarding cerebral edema, EIT has the advantages of real-time monitoring of the intracranial pressure and the evaluation of treatment effect of dehydration.

However, there are several issues to address. Fortunately, microelectrode array EIT, CCEIT, and the use of Ag/AgCl powder electrodes provide new ways to solve the problems of skull and skin contact impedance. However, we must also fully and comprehensively consider anatomically realistic geometry, inhomogeneity, cranium completeness, anisotropy and skull type, etc., in improving the accuracy of EIT modeling. Additionally, reconstruction algorithms need to be improved for applications in various diseases, such as with SB-IBCD, which is most effective in unveiling small intracranial conductivity changes.

In summary, the establishment of EIT as a mature and routine diagnostic technique will necessitate the accumulation of more supporting evidence from various disease models to obtain useful diagnostic information.

## Data Availability

Not applicable.

## Abbreviations

BPN: Benzylpenicillin
CCEIT: Capacitively coupled electrical impedance tomography
CSF: Cerebrospinal fluid
CT: Computed tomography
ECoG: Electrocorticogram
EEG: Electroencephalography
EIT: Electrical impedance tomography
FCCM: Four-concentric circle model
FEM: Finite element model
FMMU: Fourth Military Medical University
GPU: Graphics processing unit
HASU: Hyper Acute Stroke Unit
ICH: Intracranial hemorrhage
IISs: Interictal spikesISM: Inhomogeneous skull model
LADMM: Linearized alternating direction method of multipliers
LFP: Local field potential
MFEIT: Multifrequency electrical impedance tomography
MREIT: Magnetic resonance electrical impedance tomography
MRI: Magnetic resonance imaging
PDIPMs: Primal–dual interior-point methods
PET: Positron emission tomography
PPM: Piecewise processing method
SB: Spilled Bregman
SEEG: Stereo-EEG
SNR: Signal-to-noise ratio
SPM: Statistical parametric mapping
SPR: Signal processing-prior information-based reconstruction
SWDs: Spike-and-wave discharges
TV: Total variation
UCL: University College London
UCLH: UCL Hospital
VPL: Ventral posterolateral
